# Role of *AtCPK5* and *AtCPK6* in the regulation of the plant immune response triggered by rhamnolipids in Arabidopsis

**DOI:** 10.1371/journal.pone.0346370

**Published:** 2026-04-13

**Authors:** Juliette Stanek, Olivier Fernandez, Marie Boudsocq, Dina Aggad, Sandra Villaume, Laetitia Parent, Sandrine Dhondt-Cordelier, Jérôme Crouzet, Stéphan Dorey, Sylvain Cordelier

**Affiliations:** 1 Université de Reims Champagne Ardenne, INRAE, RIBP, UMR 1488, Reims, France; 2 Université Paris Saclay, CNRS, INRAE, Université Evry, Université Paris Cité, Institute of Plant Sciences Paris-Saclay IPS2, Gif sur-Yvette, France; 3 Plateau Technique Mobile en Cytométrie Environnementale MOBICYTE, UFR Sciences Exactes et Naturelles, Université de Reims Champagne-Ardenne/INERIS, Reims, France; University of Agriculture Faisalabad, PAKISTAN

## Abstract

Rhamnolipids (RLs) are bacterial glycolipids with potential applications in the biocontrol of plant pathogens. Although RLs are known to activate plant immune responses, the underlying signaling mechanisms remain poorly understood. Calcium-dependent protein kinases (CPKs) are a large family of kinases involved in various functions in plants including signaling of the plant immunity. Here, we investigated the contribution of *At*CPK5 and *At*CPK6 to RL-triggered immunity in Arabidopsis. RL treatment induced the expression of both *AtCPK5* and *AtCPK6* genes in Arabidopsis leaves. Functional analyses revealed that RL-induced responses, including reactive oxygen species production and the expression of defense-related genes (*AtWRKY46*, *AtFRK1* and *AtPR1*), were enhanced in *cpk5/6* mutants compared to wild-type plants. The *cpk5* mutant exhibited intermediate responses, whereas *cpk6* alone had little effect, except on *AtFRK1* expression, indicating a predominant role for *At*CPK5 in regulating RL-triggered signaling. However, *cpk5/6* mutations did not affect RL-induced electrolyte leakage or RL-mediated resistance to *Pseudomonas syringae* pv. *tomato* DC3000. Together, these results suggest that *At*CPK5 and *At*CPK6 negatively modulate RL-triggered immune signaling, while additional components contribute to the regulation of downstream defense responses.

## Introduction

Under natural conditions, plants are subject to various biotic stresses such as pathogen attacks [1,2]. In order to overcome these aggressions, they are able to activate defense mechanisms, which are largely based on the activation of an effective immune response [3–5]. As a first key step of this immune response, plant cells detect pathogens through the recognition of Invasion Patterns (IPs; [6]) by Pattern Recognition Receptors (PRRs) activating the so-called Pattern-Triggered Immunity (PTI; [7]). Following this recognition phase, different signaling events occur, including the production of Reactive Oxygen Species (ROS), phosphorylation/dephosphorylation cascades and ion fluxes [3,8,9]. The influx of calcium ions (Ca^2+^) represents one of the key signaling events in plant immune responses. Transient cytosolic [Ca^2+^] elevations function as secondary messengers translating pathogen perception into appropriate defense responses [10,11]. When a given stress condition arises, a significant release of Ca^2+^ ions occurs into the cytosol. Additionally, a specific Ca^2+^ signature can be associated to specific biotic and abiotic stresses [12–14]. These ions are detected by various Ca^2+^ sensors such as calmodulins (CaMs), calcium and calmodulin-dependent protein kinases (CCaMKs), calcineurin B-like proteins (CBLs) and calcium-dependent protein kinases (CPKs) [12,15,16]. Ca^2+^ modifies the structural conformation and/or enzymatic activity of these Ca^2+^ sensors, which activates target proteins to transfer signals to downstream pathways.

CPKs are involved in a wide range of functions, including plant development (roots and shoots) and plant responses to biotic and abiotic stresses [17,18]. In Arabidopsis, there are 34 different CPKs [19]. CPKs share a four-part structure including a variable N-terminal domain, a Serine/Threonine protein kinase domain, an auto-inhibitory junction domain and a C-terminal calmodulin-like domain (EF-hand domain). Their activation is triggered by the binding of cytosolic Ca^2+^ to their EF-hand domains [20]. Based on sequence similarity, particularly within the catalytic kinase domain and the regulatory calmodulin-like domain, CPKs are phylogenetically classified into four subgroups (I-IV). This classification may reflect functional diversification and evolutionary adaptation to different signaling pathways [21,22]. CPKs functions can be quite distinct from one sub-group to another. To date, 10 CPKs have been shown to be involved in biotic stress responses in Arabidopsis [23]. For example, *At*CPK1 acts as a positive regulator of resistance against pathogens with different lifestyles, such as *Botrytis cinerea* or *Pseudomonas syringae* pv.*tomato* DC3000 (hereafter referred to as *Pst* DC3000; [24]). Both *At*CPK4 and *At*CPK11, are known to act as positive regulators of pathogen responses during Effector-Triggered Immunity (ETI) and PTI by regulating defense gene expression and *At*RBOHD phosphorylation [17,25]. Several authors have emphasized the redundant action of *At*CPK5 and *At*CPK6 under biotic stress conditions. They are involved in phosphorylation of transcription factors like WRKYs and the induction of antimicrobial metabolites such as camalexin [26,27]. The Arabidopsis loss-of-function double mutant *cpk5/6* showed reduced resistance to pathogens like *B. cinerea* [28,29]*, Pst DC3000, Pst avrRpm1* and *Pst avrRpt2* [26,30], highlighting their redundant and essential roles in PTI and ETI. They are also involved in Programmed Cell Death (PCD) and ethylene biosynthesis in response to *B. cinerea* [27–29].

Over the last decade, amphipathic IPs, such as glycolipids and lipopeptides, have been investigated as novel natural bio-based molecules for the biocontrol of plant diseases [31,32]. Rhamnolipids (RLs) are natural, highly biodegradable molecules that can induce disease resistance to phytopathogens in various plant species [33]. Natural RLs activate an immune response in grapevine, rapeseed and in Arabidopsis [34–36]. In Arabidopsis, the lipid tail of RLs (HAAs, for (R)-3-hydroxyalkanoates) is perceived by the bulb-type lectin receptor kinase LIPOOLIGOSACCHARIDE-SPECIFIC REDUCED ELICITATION/S-DOMAIN-1–29 (LORE/SD1–29), which also mediates medium-chain 3-hydroxy fatty acids (mc-3-OH-FAs) sensing. On one hand, HAAs and mc-3-OH-FAs trigger an immune response exemplified by an early ROS production [37,38]. On the other hand, RLs elicit LORE-independent defense responses [38]. This RL-induced immune response is characterized by the up-regulation of classical defense genes [34,35] and in a non-canonical ROS signature displayed by a late and sustained *At*RBOHD-dependent ROS production [6]. The receptor-like cytoplasmic kinase Botrytis-Induced Kinase1 (*At*BIK1), which often regulates the activation of *At*RBOHD [39], is not involved in RL-induced response [6]. The components involved in *At*RBOHD activation following RL perception therefore remain unknown.

*At*RBOHD can also be activated by phosphorylation from subgroup I CPKs, especially *At*CPK5 and *At*CPK6 [40,41] that are known to be regulated by calcium influx and involved in a wide range of defense responses including gene expression, biosynthesis of hormones (SA and ethylene) and phytoalexins (camalexin), systemic acquired resistance, leading to resistance to multiple virulent and avirulent bacteria as well as fungi [42]. These proteins thus naturally emerged as potential candidates involved in RL-induced plant immunity, and more broadly in immune signaling triggered by amphipathic molecules. In this study, we therefore investigated the potential role of these two CPK proteins in RL-mediated plant immune signaling. First, we demonstrated that RLs trigger the expression of *AtCPK5* and *AtCPK6* in Arabidopsis leaves. Using a functional approach, we found that a synergistic effect of *At*CPK5 and *At*CPK6 negatively regulates ROS production and defense gene expression after RL perception. However, we also showed that those CPKs are not directly involved in the RL-triggered local resistance to the hemibiotrophic bacterial pathogen *Pst* DC3000. Therefore, our results demonstrate for the first time the involvement of CPKs in RL-triggered plant immune signaling, although additional components are required to establish resistance to *Pseudomonas syringae* pv. *tomato* DC3000.

## Materials and methods

### Plant material and molecules

Arabidopsis ecotype Col-0 was used as wild-type (WT) parent for all experiments. Seeds from *cpk5* (sail_657_C06), *cpk6* (salk_025460) *and cpk5/6* (sail_657_C06, salk_025460*)* Arabidopsis homozygous mutants were previously reported by Boudsocq *et al.* [30]. Seeds from *sd1−29* (*lore-5*), Col-0^AEQ^, and *lore-5*^AEQ^ Arabidopsis homozygous mutants were provided by S. Ranf [38,43]. All Arabidopsis mutants are in the Col-0 background. Plants were grown on Presstopf Tray soil (Gramoflor, Vechta Niedersachsen, Germany) in growth chambers at 20°C, under 12-h light/12-h dark regime and 60% relative humidity. RLs (AGAE® Company, USA) were used at 0,6 mg/mL in water [35,38] for all experiments.

### Conductivity assay

Conductivity assays were carried out on 5- to 6-week-old Arabidopsis plants cultured on soil in growth chamber. Four leaf discs of 6 mm diameter were incubated in distilled water for 2 hours. Two discs were placed in a well of a 12-well plate (Falcon®) containing fresh distilled water with the RLs and two in water for control. Conductivity measurements (three replicates for each treatment) were then conducted 24 hours after treatment using a B-771 LaquaTwin (Horiba) conductivity meter.

### Extra-cellular ROS production and Ca^2+^ influx analysis

ROS assays were carried out on 5- to 6-week-old Arabidopsis plants cultured on soil in growth chamber. Leaf discs of 4 mm diameter were cut and placed in 150 μL distilled water overnight in a 96-well plate (Falcon®). The following processes were made as detailed by [44]. Luminescence (Relative Light Units, RLU) was measured every 5 min during 12 hours with a TECAN SPARK Multimode Microplate Reader. Control was realized on leaf discs of WT plants with water. Ca^2+^ influx analysis were performed using Col-0^AEQ^ and *lore-5*^AEQ^ mutant as detailed by [43]. Luminescence measurements were performed following the same procedure with a TECAN SPARK Multimode Microplate Reader (TECAN®).

### Gene expression analysis

In our assays, leaf discs of 6 mm diameter of 5- to 6-week-old Arabidopsis plants were cut and placed in 2 mL distilled water for 2 hours in a 24-well plate (Falcon®). They were collected at 0 hours, 9 hours and 24 hours after RL treatment and crushed with liquid nitrogen. 50 mg of each sample were placed in 2 mL cold safe-lock Eppendorf. 1 mL of QIAzol Lysis Reagent (QIAGEN®) were dropped in each tube and were vortexed. After 5 minutes of incubation, chloroform/IAA (24:1) was added. All the samples were vigorously shaken and centrifuged for 15 minutes at 10,000 g. The aqueous phase is collected and precipitated with isopropanol then centrifuged at 10,000 g. Samples were washed with 70% ethanol and pellets were solubilized in purified water DNAse/RNAse free. cDNAs were obtained following a Reverse Transcriptase protocol (EuroBlueTaq kit).

Quantitative RT-PCR was carried out on three independent biological replicates for each sample, as well as two technical replicates for each reaction. Quantitative RT-PCRs were performed using qPCRBIO SyGreen Blue Mix Lo-Rox (Eurobio Scientific, France, Les Ulis) in white 384-well plates (Sorenson, USA, Salt Lake City) and on a CFX Opus 384 instrument (Bio-Rad, USA, Hercules). Prior to the defense gene expression level experiments, the expression stability of a set of five housekeeping genes (*AtActin7*, *AtActin2*, *AtTubulin4*, *AtUbiquitin5* and *AtUbiquitin10*) in *A. thaliana* plant leaves both treated and untreated with RLs was assessed. The M-Score calculation was performed using the BioRad CFX Maestro software to select the three most stable genes under our experimental conditions. *AtActin7* (GenBank NM_121018.4; [45], *AtActin2* (GenBank NM_112764.4; [46] and *AtTubulin4* (GenBank NC_003074.8; [47]) were subsequently selected as reference genes for normalisation. The primers used in this study are listed in S1 Table and some were previously reported ([30].

### Pst DC3000 culture and disease-resistance assays

*P. syringae* *pv.* *tomato* strain DC3000 (*Pst* DC3000) was grown at 28 °C under stirring in King’s B (KB) liquid medium supplemented with antibiotic: 50 μg/mL rifampicin. For protection assays, Arabidopsis plants were grown individually for 4 weeks in soil. For each experiment, six pots per condition were used (n = 6). The following processes were made as detailed in [38]. Two days before infection, plants were sprayed with RLs or water as control and were placed in high humidity atmosphere. Plants were then infiltrated with bacterial suspension at the concentration of 10^7^ CFU/mL (in 10 mM MgCl_2_) using a needleless syringe. Bacterial quantification *in planta* (colony forming units; CFU) was performed 3 days post infection (dpi). To this end, all plant leaves from the same pot were harvested, weighed, and crushed in a mortar with 10 mL of 10 mM MgCl_2_, and serial dilutions were performed. For each dilution, 10 μL were dropped on KB plate supplemented with appropriate antibiotics. CFU were counted after 2 days of incubation at 28 °C. The number of bacteria per milligram of plant fresh mass was obtained with the following formula:


$$CFU.{mg}^{-\text{1}}=\frac{\left(\frac{N\times Vd}{Vi}\times {\text{1}\text{0}}^{n-\text{1}}\times \text{1}\text{0}\text{0}\right)}{M}$$

with N equal to CFU number, Vi the volume depot on plate, Vd the total volume, n the dilution number, and M the plant fresh mass.

### Sample handling, biological replicates and statistical analysis

**Fig 1:**
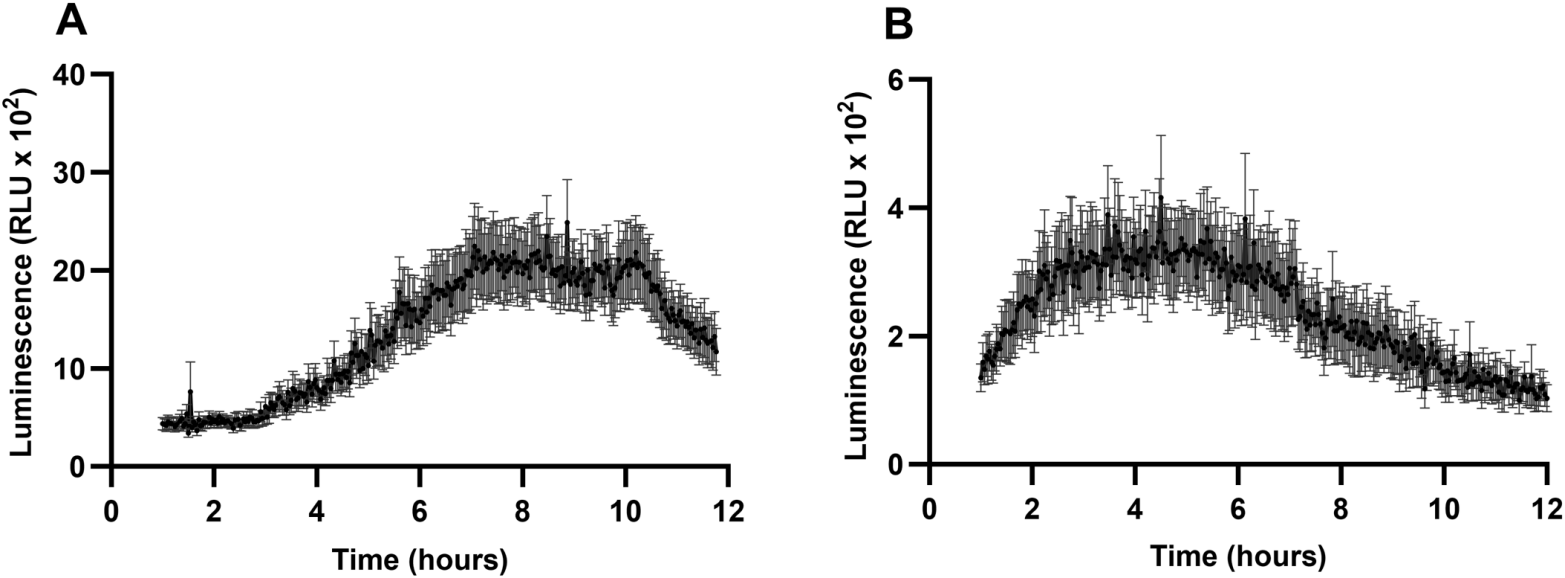
RL induce late cytoplasmic [Ca2+] production in Arabidopsis leaf. [Ca^2+^]cyt of WT-Aeq (**A**) and *lore-5*-Aeq (**B**) was quantified in Arabidopsis leaves after RL treatment (0.6 mg/mL). RLU amounts were analysed over 12 hours. Data are presented as mean ± SEM (n = 7, experiments were performed two times with similar results).

All experiments were performed in independent triplicates (*i.e.,* *A. thaliana* plants were grown under reproducible conditions but on three separate dates for each experiment) except for Fig 1 which involved aequorin mutants (two independent experiments). The number of individual plants used varied from six to eighteen, depending on the experiment (the exact number is indicated individually on each Figure) and this represents the total number of plants used in all the independent replicates.

All data were subjected to statistical analysis using various tests with the Rstudio software and the preloaded “stats” package. Additionally, the “vegan” package was used for the PCoA analysis combined with the “TSDist” package to implement Fréchet distance. PCoA can be performed using a variety of distances depending on the phenomenon being studied. The vegan package implements the Euclidean distance by default, but specialists in microbial ecology often use specific distances (such as Bray-Curtis or Jensen-Shannon), which can address the double-zero problem [48]. In our case, we chose Fréchet distance [49] for its suitability for time-dependent phenomena such as the kinetics of ROS production. GraphPad software was used for graphical representation of data.

## Results

### RLs trigger Ca² ⁺ influx in Arabidopsis leaves

We first investigated Ca^2+^ influx following RL challenge, as it is an essential component for CPK activation [50]. In order to follow the RL-induced Ca^2+^ influx, we used the Col-0^AEQ^ and *lore-5*^AEQ^ Arabidopsis homozygous mutants carrying the calcium reporter aequorin to perform the Ca^2+^ assay. RLs are known to induce a strong, late and sustained production of ROS in Arabidopsis leaves from 3 to 12 hours post treatment (hpt; [38]). Similarly, we observed a strong and late influx of Ca^2+^ in Col-0^Aeq^ Arabidopsis plant after challenge with RLs, starting at 3hpt and decreasing at 10hpt (Fig 1A). A Ca^2+^ influx was also observed in *lore-5*^Aeq^ plants that does not sense 3-OH-FA and HAA precursors, which could be found in trace amount in RL solution (Fig 1B; [38]). These results clearly demonstrate that RLs are inducing Ca^2+^ influx in Arabidopsis.

### RLs trigger up-regulation of *AtCPK5* and *AtCPK6* genes expression

**Fig 2:**
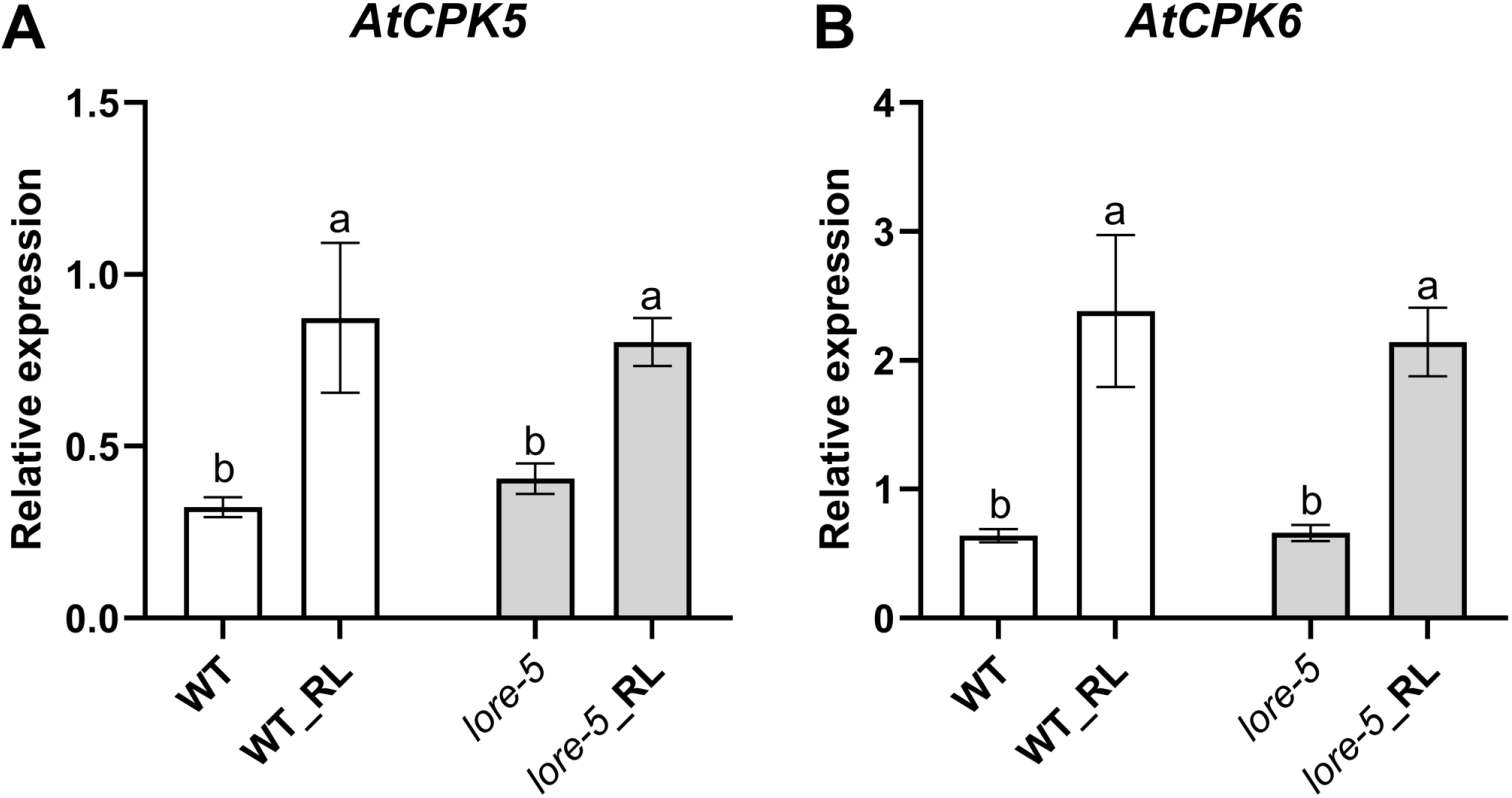
RL induce *AtCPK5* and *AtCPK6* genes expression in Arabidopsis leaf. RL induced *AtCPK5* (**A**) and *AtCPK6* (**B**) expression was studied by RT-qPCR in WT plants and *lore-5* mutants. Leaf discs were treated with RL (0.6 mg/mL) and analysed at 24 hpt. Data are presented as mean ± SEM (n = 6, three independent biological experiments). Expression data were normalized with control (non treated WT at 0 hpt) and compared with *AtActin2, AtActin7* and *AtTubulin4* as reference genes. Letters represent results of Kruskal-Wallis followed by Wilcoxon pairwise test by time, with *P* > 0.05 (same letters) or *P* ≤ 0.05 (different letters).

**Fig 3:**
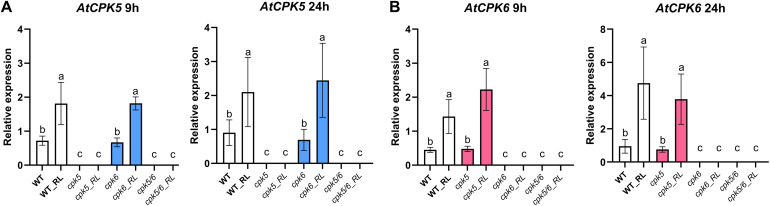
RL induce *AtCPK5* and *AtCPK6* gene expression. *AtCPK5* (**A**) and *AtCPK6* (**B**) gene expression following RL treatment was analysed by RT-qPCR in WT plants and *cpk5*, *cpk6* and *cpk5/6* mutants. Leaf discs were treated with RL (0.6 mg/mL) and analysed at 0hpt, 9 hpt and 24 hpt (0 hpt data are not presented here). Expression data were normalized with *AtActin7*, *AtActin2* and *AtTubulin4* as reference genes. Data are presented as mean ± SEM (n = 6, three independent biological experiments). Letters represent results of Kruskal-Wallis followed by Wilcoxon pairwise test by time, with *P* > 0.05 (same letters) or *P* ≤ 0.05 (different letters).

*AtCPK5* and *AtCPK6* gene expression was examined in WT and in *lore-5* plants after treatment with RLs (Fig 2). RT-qPCR results show a significant increase in transcripts of *AtCPK5* and *AtCPK6* is both WT and mutant plants 24h after RL challenge with no significant differences in both plant backgrounds (Fig 2A, 2B). The relative expression of *AtCPK5* at 9 hpt and 24 hpt in WT, *cpk5*, *cpk6* and *cpk5/6* plants revealed that the gene was induced to a similar extent in WT and in *cpk6* mutants following RL treatment (Fig 3A). As expected, no expression of *AtCPK5* was observed in the *cpk5* and *cpk5/6* mutant plants. Similarly, there was no significant difference in *AtCPK6* gene expression levels between WT plants and *cpk5* mutant plants both treated with RLs (Fig 3B). No *AtCPK6* expression was detected in both *cpk6* and *cpk5/6* plants.

### Extracellular ROS production is increased in *cpk5/6* background following RL perception

**Fig 4:**
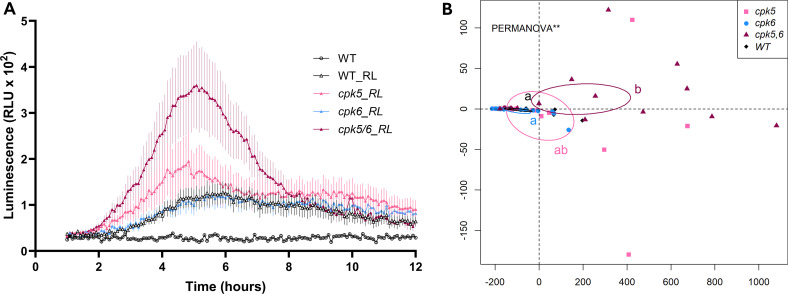
CPK5 and CPK6 are involved in ROS production after RL treatment in Arabidospis leaf. **(A)** Extracellular ROS production after treatment of WT, *cpk5*, *cpk6* and *cpk5/6* mutants leaf discs with 0.6 mg/mL RL or water as control. ROS production was analysed from 1 hpt to 12 hpt. **(*B*)** Principal Coordinate Analysis (PCoA) plot deduced from Fréchet distances between temporal measurements of ROS production during the second peak following RL treatment. The PERMANOVA test (and its pairwise version using FDR correction) was used for statistical analysis. Different letters indicate statistically different groups (p < 0.01). Ellipses display confidence intervals (95%). **(*A*–*B*)** Data are mean ± SEM (*n* = 18, three independent biological experiments).

We compared ROS production levels after RL treatment from 1 to 12 hpt (corresponding to the typical RL-triggered ROS signature; [38] in WT and mutant backgrounds (Fig 4A). ROS production in *cpk6* leaves was similar to WT leaves, while a little higher in *cpk5*. However, the production of extracellular ROS was strongly enhanced in the *cpk5/6* double mutant leaves (Fig 4A). To complete our statistical analysis of the ROS production, we performed a PCoA analysis, based on the calculation of the Frechet distance between each measurement for each condition at each time point in the series. The following PERMANOVA analysis on the resulting intergroup distance allowed us to conclude that only the *cpk5/6* double mutant exhibited significantly elevated levels compared to WT (Fig 4B). This analysis indicates that both *At*CPK5 and *At*CPK6 negatively regulate the ROS burst induced by RLs, with *At*CPK5 likely playing a major role.

### *At*CPK5 and *At*CPK6 are involved in regulation of defense genes activated by RLs

To determine whether *At*CPK5 and *At*CPK6 would potentially be involved in the regulation of defense genes following RL challenge, we analysed the expression of *AtWRKY46* (NM_130204.3) and *AtFRK1* (Flg22-Induced Receptor-Like Kinase 1, NM_127476.2) as early defense markers known to be involved in classical PTI [51,52] and *AtPR1* (Pathogenesis-Related 1, NM_127025.3) as a late defense marker. Defense-related gene markers commonly used to monitor pattern-triggered immunity (PTI) are also routinely used to analyze transcriptional responses induced by RLs [35]. While *AtWRKY46* gene has been shown to regulate the plant defense mechanisms [53,54], *AtFRK1* is a well-established defense marker gene induced by classical elicitors such as flg22 and chitin [55], as well as by RL precursors [37] and is therefore widely used as a marker of plant immune activation [56]. *AtPR1* is a classical defense-related gene that is widely used as a molecular marker for the salicylic acid signaling pathway, and resistance to biotrophic pathogens [57–60].

**Fig 5:**
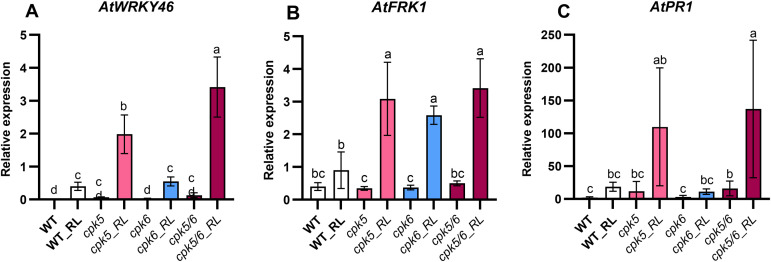
*AtCPK5* and *AtCPK6* are involved in early and late defense gene expression after RL treatment. *AtWRKY46* **(A)**, *AtFRK1* (**B**) and *AtPR1* (**C**) gene expression was studied by RT-qPCR in WT plants and *cpk5*, *cpk6* and *cpk5/6* mutants. Leaf discs were treated with RL (0.6 mg/mL) and analysed at 9 hpt for early defense gene expression (*AtWRKY46* and *AtFRK1*) and at 24 hpt for late defense gene expression (*AtPR1*). Data are presented as mean ± SEM (n = 6, three independent biological experiments). Expression data were normalized with *AtActin7*, *AtActin2* and *AtTubulin4* as reference genes. Letters represent results of Kruskal-Wallis followed by Wilcoxon pairwise test by time, with *P* > 0.05 (same letters) or *P* ≤ 0.05 (different letters).

*AtWRKY46* gene was slightly upregulated at 9 hpt in RL-treated WT plants (Fig 5A). Compared to WT, a 3 and 6-fold increase in *AtWRKY46* expression was respectively observed in *cpk5* and *cpk5/6* mutants following RL challenge. No difference was found in *AtWRKY46* induction between WT and *cpk6* mutant (Fig 5A). However, *At*CPK6 also appears to be involved, since *AtWRKY46* is more highly expressed in the *cpk5/6* double mutant than in the *cpk5* single mutant. Following RL treatment, the expression pattern of *AtFRK1* gene was similarly increased in *cpk5, cpk6* and *cpk5/6* mutants (Fig 5B). *AtPR1* expression was slightly up-regulated in the *cpk5* mutant after RL challenge, although not statistically significant, but not in *cpk6* mutant (Fig 5C). However, *At*CPK6 also appears to be involved, since *AtPR1* is statistically more highly expressed in *cpk5/6* than in WT.

### *AtCPK5* and *AtCPK6* inactivation does not affect RL-triggered electrolyte leakage

**Fig 6:**
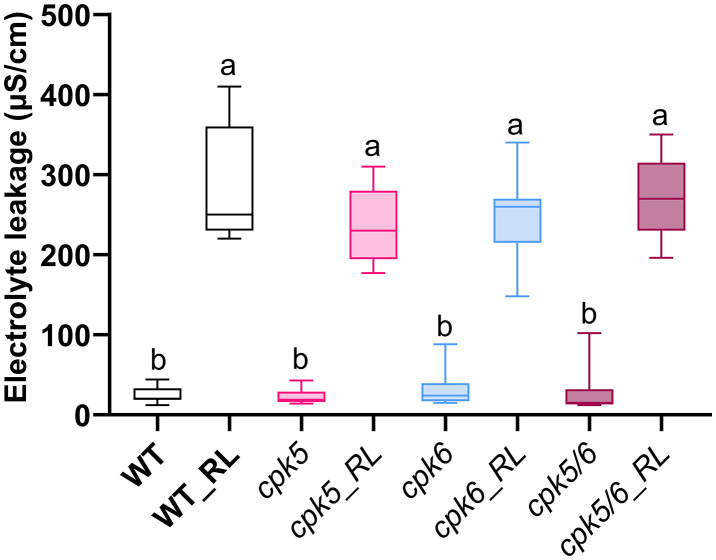
*AtCPK5* and *AtCPK6* are not involved in RL-induced electrolyte leakage on Arabidopsis leaf. Electrolyte leakage was measured on WT, *cpk5*, *cpk5* and *cpk5/6* Arabidopsis leaf discs 24 hpt by RL (0.6 mg/mL) or water (control). Data are mean ± SEM (n = 9, three independent biological experiments). Letters represent results of Kruskal-Wallis followed by Wilcoxon pairwise test by time, with *P* > 0.05 (same letters) or *P* ≤ 0.05 (different letters).

Electrolyte leakage is a typical marker of RL-triggered immunity [38]. As expected, when treated with RLs, WT plants displayed a strong increase of electrolyte leakage at 24 hpt (Fig 6). This response was conserved in *cpk5*, *cpk6* and *cpk5/6* mutant plants, which showed no significant differences compared to WT plants.

### *At*CPK5 and *At*CPK6 are not involved in Arabidopsis RL-triggered resistance to the hemibiotrophic pathogen *Pst* DC3000

**Fig 7:**
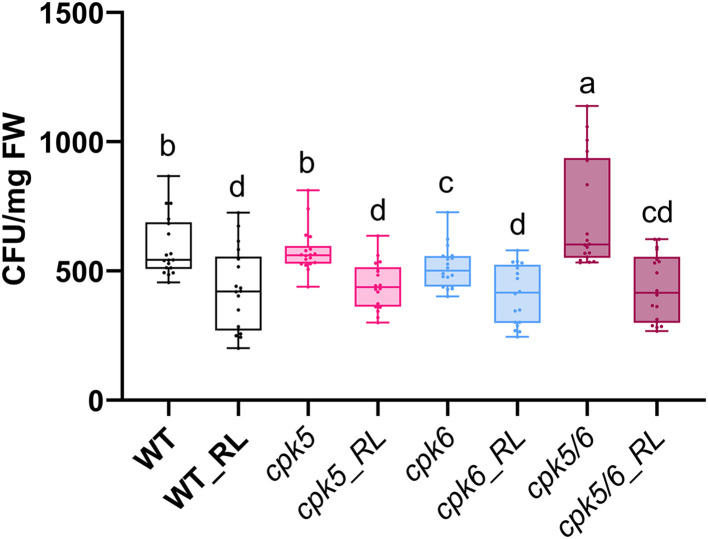
*AtCPK5* and *AtCPK6* are not involved in RL-induced *Pst* protection on Arabidopsis leaf. WT, *cpk5, cpk6* and *cpk5/6* Arabidopsis leaves were treated with RL (0.6 mg/mL) or water (control) 48 h before infection. *Pst* colonies were counted at 3 dpi. Data are individual data ± SEM (*n* = 18, three independent biological experiments). Letters represent results of Kruskal-Wallis followed by Wilcoxon pairwise test by time, with *P* > 0.05 (same letters) or *P* ≤ 0.05 (different letters).

*Pst* DC3000 is a well-studied model pathogen and is classified as a hemibiotrophic pathogen that initially feeds on living plant tissues and later causes the death of plant cells [61–63]. To study RL-induced resistance to *Pst* DC3000, Arabidopsis plants were pre-treated with water control or RLs 2 days before bacterial infection. In control conditions, the *cpk5* and *cpk6* single mutants display a sensitivity to *Pst* DC3000 comparable to the WT while the *cpk5/6* double mutant was hypersensitive (Fig 7), as previously reported [30]. Upon RL treatment, all plant backgrounds, including the three *cpk* mutants, displayed the same level of protection induced by RL treatment against *Pst* DC3000, indicating that *At*CPK5 and *At*CPK6 are not essential for RL-triggered resistance (Fig 7).

## Discussion

In this study, we demonstrated that RLs activate Ca^2+^ influx in Arabidopsis. This is the first time that RLs have been shown to induce a sustained and late Ca^2+^ signature in Arabidopsis. This signature is similar to analyses of extracellular ROS production following RL challenge [38]. Interestingly, previous studies have suggested that these two types of signatures may be interconnected [64,65]. It has been shown that Ca^2+^ is important at different levels of plant immunity. Transient Ca^2+^ fluxes, mediated by Ca^2+^ permeable channels and decoded by Ca^2+^ binding sensor proteins, regulate various downstream cellular processes that are essential for both NLR- and PRR-mediated immunity [65,66]. The late and long-lasting Ca^2+^ signature is quite singular for a biotic elicitor since, for the majority of MAMPs (Microbe-Associated Molecular Patterns) characterized to date, it has been shown that the Ca^2+^ influx is an early process (during the first few minutes) in plant immunity [67]. Interestingly, biphasic cytosolic calcium signatures have been widely reported in plant responses to diverse pathogens and MAMP. The kinetics and amplitude of each phase could vary depending on the MAMP nature, suggesting that a late and long signature is more reminiscent to ETI or some abiotic stresses [68]. In the context of RL signaling, the initial transient calcium peak may be triggered by RL precursors [37], whereas the subsequent sustained calcium peak could result from the amphipathic properties of RLs themselves (Fig 1B). Recent studies indicate that amphipathic molecules such as RLs and lipopeptides are perceived through unconventional mechanisms involving plant plasma membrane lipids, which may underlie their distinctive immune signaling activity [32]. Given their amphipathic nature, it has been proposed that RLs interact directly with membrane lipids [35,38]. Supporting this hypothesis, rhamnolipids were shown to insert into plant lipid-based membrane models [69]. In parallel, lipopeptides were proposed to stimulate plant immunity primarily through alterations in membrane mechanical properties rather than membrane permeabilization [70]. Consistently, amphipathic sphingolipids were shown to disturb plasma membrane organization, thereby affecting effector localization and function [71]. Together, these observations support the hypothesis that amphipathic molecules, by perturbing plasma membrane organization, may induce a delayed cytosolic calcium influx through the activation of plasma membrane–localized mechanosensitive calcium channels or calcium transporters. This late calcium signal could in turn regulate CPK activity, thereby contributing to downstream immune signaling.

The expression of the two *AtCPK5* and *AtCPK6* genes was analyzed at 9 h, corresponding to a time point shortly after the calcium and ROS peaks (Figs 1 and 4), and at 24 h, a later time point commonly used to study gene expression involved in RL responses [34–36]. Consistent with the role of Ca^2+^ influx and ROS production in RL signaling, we found that RLs induce the expression of *AtCPK5* and *AtCPK6* genes. These CPKs are already known as positive regulators of plant immunity in response to various bacteria and fungi, regulating ROS burst, gene expression and hormone signaling [26–28,30,72].

RLs also activate an atypical ROS production signature in plants [38,73]. When ROS production was monitored in the three CPK-related mutants (*cpk5, cpk6 and cp5/6*) and compared to the parental line WT, it appeared that the most significant difference with WT was observed in *cpk5/6*, suggesting that both *At*CPK5 and *At*CPK6 could act in synergy, but potentially as negative regulators. Other CPKs, such as *At*CPK8, have also been shown to negatively regulate oxidative stress. The *cpk8* mutant accumulated more ROS in leaves and stomatal guard cells compared with WT plants when treated with abscisic acid (ABA) [74]. *At*CPK28 was also described as a negative regulator of MAMP-induced oxidative burst, increasing the turnover of Botrytis-Induced Kinase1 (BIK1; [75]), an enzyme that activates *At*RBOHD. However, the negative effect observed here has to be indirect since *At*CPK5 and *At*CPK6 have been shown to activate *At*RBOHD-mediated ROS production by direct phosphorylation of the NADPH oxidase, in response to flagellin 22 (flg22) [40,41].

A positive regulation by *At*CPK5/6 homologs is also found in other plant species. For example, *Os*CPK5/*Os*CPK13 and *St*CPK4/*St*CPK5 act as positive regulators of ROS production via *Os*RBOHD/*St*RBOHB phosphorylation in *Oryza sativa* and *Solanum tuberosum*, respectively [76,77]. Interestingly, it was recently reported that, despite the positive role of *Os*CPK5/*Os*CPK13 in PTI responses, the double mutant was more resistant to rice blast and displayed increased ROS production and marker gene expression *OsPR5* and *OsWRKY45* [78]. The NLR *Os*CARP1 (*Os*CPK5/13-Associating Resistance Protein 1) was shown to interact with *Os*CPK5/*Os*CPK13 and was required for the enhanced blast resistance of *oscpk5/oscpk13*. Moreover, *Os*CARP1-induced cell death was suppressed by the co-expression with both CPKs. This led the authors to propose that the two protein kinases were guarded by the NLR. We could thus hypothesize that a similar mechanism occurs in Arabidopsis: RL perception could activate a still unknown NLR that guards *At*CPK5/*At*CPK6. In the absence of both kinases, the NLR would trigger ETI, including ROS burst and defense gene expression.

In line with this model, we also observed that RL-triggered activation of early and late defense genes was affected by *AtCPK5* and *AtCPK6* inactivation. Indeed, the expression of *AtWRKY46* and *AtPR1* was significantly higher in *cpk5/6* mutants compared to WT while the three treated mutants showed similar increased levels of *AtFRK1* expression. Besides, this could also support the hypothesis that *At*CPK5 and *At*CPK6 may function as negative regulators of defense gene expression in response to RLs. Indeed, although they have previously been identified as redundant positive regulators of defense gene expression in response to different MAMPs and Damage-Associated Molecular Patterns, such as flg22, elongation factor 18 (elf18), plant elicitor peptides 3 (PEP3), and oligogalacturonides (OGs), most commonly through the regulation of WRKY transcription factors [25–28,30], a recent report suggested a negative regulation for some genes depending on growth conditions. It was shown that the glucose analog 2-deoxyglucose (2DG) can influence plant’s defense and AtCPK5/6 were involved in this process [79]. The 2DG-induction of defense-related genes was differentially altered in *cpk5/6* showing decrease of *AtNHL10* (NDR1/HIN1-Like 10) and *AtPAD3* (Phytoalexin Deficient 3) induction but increase of the salicylic acid biosynthesis genes *AtPBS3* (AvrPphB Susceptible 3) and *AtSID2* (Salicylic acid Induction-Deficient 2), indicating the versatility of this type of kinases. As a result, *cpk5/6* accumulated more SA in response to 2-DG although AtCPK5 was shown to promote SA biosynthesis and signaling [40,80]. This suggests that AtCPK5/6 may differentially regulate defense responses depending on growth conditions. Since *AtWRKY46, AtFRK1* and *AtPR1* genes are positively regulated by SA [53,81,82], their increased induction observed in *cpk5, cpk6* and/or *cpk5/6* following RL treatment may result from SA accumulation. Several reports have established that *At*CPK5 and *At*CPK6 play a pivotal role in modulating phytohormone biosynthesis, including the SA and ethylene pathways [28,40,83,84]. Collectively, these findings suggest that *At*CPK5/6 could act as components of Ca^2+^ signals to coordinate hormonal regulation of some defense gene expression in Arabidopsis following RL perception*.*

*At*CPK5 and *At*CPK6 have previously been shown to act together since some phenotypes are detected only in the double mutant. For instance, they function as positive, redundant regulators of *B. cinerea*-induced camalexin biosynthesis [27]. Both CPKs are required for the resistance to *Pst*DC3000 [30] and to *Psm* ES 4326 after priming with the avirulent strain *Psm avrRpm1* [80]. Nonetheless, some reports also highlighted a specific or major role for *At*CPK5. The constitutive immune phenotype of *exo70B1* mutant can be reverted specifically by *cpk5*, but not *cpk6,* and depends on the truncated NBS-NLR TN2 which interacts with AtCPK5 but not AtCPK6 [85]. The flg22-induced phosphorylation of *At*RBOHD was already reduced in single *cpk5,* leading to reduced oxidative burst [40]. The systemic induction by *Psm avrRpm1* of some marker genes like *NHL10* was reduced in both *cpk5* and *cpk5,6* but not *cpk6* [80]. Under our experimental conditions, ROS production in response to RLs was slightly higher in *cpk5* than in WT, while *cpk6* displayed WT response. Similarly, *AtWRKY46* and *AtPR1* were already induced in *cpk5*, but not *cpk6* upon RLs treatment. Thus, although both CPKs are involved in plant immunity, *At*CPK5 can exhibit a major role under some growth conditions, or through the specific interaction with partners or substrates.

The *cpk5/6* double mutant exhibits increased susceptibility to the pathogenic bacteria *Pst* DC3000, indicating that *At*CPK5 and *At*CPK6 act as positive regulators of basal resistance against this bacterial infection. Our findings are consistent with previous reports showing that these kinases contribute in duo to effective plant defense responses [30]. In the context of RL challenge, a protective effect against *Pst* DC3000 was clearly confirmed in WT plants, as previously demonstrated [38]. Interestingly, this resistance effect was not abolished in *cpk5*, *cpk6* or *cpk5/6* mutant lines. Therefore, although our data support the involvement of *At*CPK5 and *At*CPK6 in RL-triggered signaling events and some defense responses, the RL-induced local resistance is not affected. This disconnexion between molecular responses and pathogen resistance has already been reported for *cpk5/6* in response to *Pst* DC3000(ΔavrPtoΔavrPtoB) [79]. One explanation could be the involvement of other CPKs such as *At*CPK1/2/4/11 that are also key regulators of immune responses [24,26,30,35,86,87], or entirely distinct branches of the immune network. Moreover, we cannot rule out the possibility that subtle or transient effects have not been captured in our experimental conditions. Direct resistance to a pathogen can differ from induced resistance triggered by specific elicitors. For example, RLs are known to activate both SA and JA/ethylene pathways in Arabidopsis, thereby triggering a resistance to necrotrophic and biotrophic pathogen agents [35]. In this case, the classical dichotomy between pathogen with different lifestyles and phytohormones is not conserved.

Various studies have shown that RLs integrate into the plasma membrane in a manner reminiscent of mechanical stresses [33,69]. Consequently, it is plausible that the perception of RLs, despite their bacterial origin, bear similarities with the perception of an abiotic stress. Electrolyte leakage is commonly used to monitor plasma membrane damage and structural perturbation, which can sometimes lead to cell death. Because RLs can insert into plasma membranes and alter their structure [88], electrolyte leakage measurements were used to assess a potential link between plant plasma membrane perturbation and RL-induced signaling involving CPKs. Accordingly, RLs induce electrolyte leakage in Arabidopsis cells (Fig 6 and [38]). The lack of significant differences among the analyzed genotypes suggests that RL-induced electrolyte leakage is not directly associated with CPK-dependent signaling. It has been shown that WT plant roots induce a late peak (6 hours after treatment) of ROS after the induction of salt stress [89]. This is consistent with the ROS production we observed after RL challenge. Several CPKs have previously been identified as negative regulators of abiotic stress responses. For instance, *At*CPK9 and *At*CPK33 negatively regulate ABA-induced stomatal closure by modulating anionic currents [90,91], while *At*CPK21 has been described as a negative regulator of osmotic stress tolerance [92]. This functional diversity among CPKs may help to explain why *At*CPK5 and *At*CPK6 could act differently in this context compared to their well-established roles in direct biotic stress responses. Instead, electrolyte leakage following RL treatment may result from membrane permeabilization due to RL insertion or from interactions with membrane microdomains, potentially leading to the formation of resistosome-like complexes, although further investigation will be required to clarify these mechanisms [93,94].

In conclusion, in this study, we demonstrated that *At*CPK5 and *At*CPK6 can act as negative regulators of some RL-mediated immune responses including ROS production and some defense gene activation. Interestingly, despite these molecular changes, *AtCPK5* and *AtCPK6* inactivation did not change the resistance to a biotrophic pathogen, suggesting that other factors are involved in RL-mediated plant resistance. These findings highlight the complexity of *At*CPK5 and *At*CPK6 and more generally CPK networks in plant immunity and provide a basis for further studies aiming to identify complementary regulators involved in RL-triggered plant resistance. In particular, identifying substrates of *At*CPK5/6 in response to RLs will clarify whether their apparent negative role on ROS production and gene expression is direct or indirect.

## Supporting information

S1 Table Sequences of primers used for qRT-PCR analyses. F: sequence of forward primer; R: sequence of reverse primer.
(DOCX)

S1 File Raw data set used to produce Figs 1–7. Each sheet named Figs 1–7 of this Excel file contains the raw data set used to produce the corresponding Figures presented in the core manuscript.
(XLSX)

S2 File Script used to generate PERMANOVA and pairwise post-hoc tests. (DOCX)
