# Supplementary material for: Role of AtCPK5 and AtCPK6 in the regulation of the plant immune response triggered by rhamnolipids in Arabidopsis
Source: PLoS One. 2026 Apr 13;21(4):e0346370. doi: 10.1371/journal.pone.0346370 (PMC13075711; doi:10.1371/journal.pone.0346370)
Supplement: S1 Table — F: sequence of forward primer; R: sequence of reverse primer. (DOCX) [file pone.0346370.s001.docx]

| **Gene name** | **AGI Number** | **Primer sequence** |
| --- | --- | --- |
| *AtCPK5* | At4g35310 | F : TATGGATGCGGCTGATGTAG  R : GCTCTTCCCGCTCTAGTTTG |
| *AtCPK6* | At2g17290 | F : AGACAACGATGGACGGATTG  R : CTCCCTACACCAGCATTTCC |
| *AtFRK1* | At2g19190 | F : CGGTCAGATTTCAACAGTTGTC  R : AATAGCAGGTTGGCCTGTAATC |
| *AtPR1* | At2g14610 | F : AACTACGCTGCGAACACGTG  R : TCACTTTGGCACATCCGAGTC |
| *AtWRKY46* | At2g46400 | F : CGTGCATCTGTAATATGCTCTAGG  R : GATGATGGTCACTGCTGGAG |
| *AtACT2* | At3g18780 | F : TCCGCTCTTTCTTTCCAAGCTCA  R : CCCTGGGAGCATCATCTCCTG |
| *AtACT7* | At5g09810 | F : CCCAGGAATTGCTGACCGTA  R : TTTTCTCTCTGGCGGTGCA |
| *AtTUB4* | At5g44340 | F : AGGGAAACGAAGACAGCAAG  R : GCTCGCTAATCCTACCTTTGG |

**S1 Table :** Sequences of primers used for qRT-PCR analyses.

F : sequence of forward primer; R: sequence of reverse primer
