# Supplementary material for: Role of AtCPK5 and AtCPK6 in the regulation of the plant immune response triggered by rhamnolipids in Arabidopsis
Source: PLoS One. 2026 Apr 13;21(4):e0346370. doi: 10.1371/journal.pone.0346370 (PMC13075711; doi:10.1371/journal.pone.0346370)
Supplement: S2 File — (DOCX) [file pone.0346370.s003.docx]

####Script to generate PERMANOVA and pairwise post-hoc tests (Stanek et al.) Fig 4B ####

#The following script can be used with R version 4.4.2 or earlier since

#TSdist and TSclust do not work with R version 4.5

####Package installation####

install.packages('devtools')

install.packages("vegan")

install_github("pmartinezarbizu/pairwiseAdonis/pairwiseAdonis",force=TRUE)

install_github("cran/TSdist",force=TRUE)

install_github("cran/longitudinalData")

install_github("cran/TSclust")

install.packages('TSdist')

install.packages(c('longitudinalData', 'TSclust'))

####Library uploading####

library(longitudinalData)

library(TSclust)

library(TSdist)

library(vegan)

library(pairwiseAdonis)

library(devtools)

####Uploading file ROS_kinetics_Fig4A.csv####

#This file contains the data used to generate Fig. 4A and to calculate PCoA and PERMANOVA

#Only the second peak is displayed on fig 4A (starting at t60 min = 1 hour, column 14 in the file)

#Only the second peak is used for PCoA and PERMANOVA calculations (starting at t60 min = 1 hour, column 14 in the file)

read.csv("ROS_kinetics_Fig4A.csv",row.names=1,sep=";",header=T,stringsAsFactors = T)->ROS

#Reorganising Line as a factor

ROS$Line<-factor(ROS$Line, levels=c("cpk5","cpk6","cpk5,6","WT"))

ROS_group<-ROS$Line

#Trimming the ROS data.frame to retain only the data from the second peak

ROSd<-t(ROS[,14:146])

####Frechet distance calculation####

#As explained in the article, we replaced the Euclidean distance with the Frechet distance in order to generate the PCoA.

paires_colonnes <- combn(ncol(ROSd), 2, simplify = FALSE)

ROS_dist <- sapply(paires_colonnes, function(p) {

TSDistances(ROSd[, p[1]], ROSd[, p[2]],distance="frechet")

})

#Converting the distance matrix produced by TSdist to a distance matrix compatible with vegan

n <- ncol(ROSd)

ROS_fdist <- matrix(0, n, n)

for (i in 1:length(ROS_dist)) {

pair <- paires_colonnes[[i]]

ROS_fdist[pair[1], pair[2]] <- ROS_dist[i]

ROS_fdist[pair[2], pair[1]] <- ROS_dist[i]

}

as.dist(ROS_fdist)->ROS_fdist

####Performing vegan PCOA with frechet distance####

PCOA_ROS <- cmdscale(ROS_fdist,k = 5, eig = T)

#Create the matrix of coordinate to produce graph

PCOA_ROS_points <- as.data.frame(PCOA_ROS$points)

names(PCOA_ROS_points)[1:5] <- paste0("PCoA", seq(1,5))

####Performing PERMANOVA test and pairwise Line comparison####

adonis2(ROS_fdist~ROS_group)

#Result Significant level ="**"

#Pairwise comparison with FDR correction

pairwise.adonis(ROS_fdist,ROS_group)

#Letters for significant group

#WT = a, cpk6= a, cpk 5= ab, cpk5,6= b

####Final production of Fig. 4B####

#Completing the ROS data.frame with graphical informations

rep(c(18,15,16,17),each=18)->A

rep(c("black","#FF69B4", "#1E90FF","#8B0A50"),each=18)->B

cbind(PCOA_ROS_points,A,B)->PCOA_ROS_points

#PCoA Graph

x11()

plot(PCOA_ROS_points[,1],PCOA_ROS_points[,2],pch=PCOA_ROS_points$A,col=PCOA_ROS_points$B,ylab="",xlab="")

text(PCOA_ROS_points[,1]-0.5, PCOA_ROS_points[,2]-0.5,labels="")

legend("topright",legend = c(expression(italic("cpk5")), expression(italic("cpk6")), expression(italic("cpk5,6")), expression(italic("WT"))),pch = c(15,16,17,18), col=c("#FF69B4", "#1E90FF","#8B0A50","black"))

abline(h = 0, v = 0, lty = 2)

ordiellipse(PCOA_ROS, ROS_group, col=c("#FF69B4", "#1E90FF","#8B0A50","black"),

kind = "se", lty=c(1), conf = 0.95, alpha = 0.05, lwd = 1.5)

#Adding PERMANOVA and pairwise PERMANOVA informations to the PCoA graph

text(-100,100,"PERMANOVA**")

text(450,25,"b",col="#8B0A50",cex=1.3)

text(-25,-15,"a",col="#1E90FF",cex=1.3)

text(100,-50,"ab",col="#FF69B4",cex=1.3)

text(-50,15,"a",col="black",cex=1.3)

####Remove unnecessary objects####

rm(i,n,pair,ROS_dist,paires_colonnes,ROS,ROSd,ROS_group,PCOA_ROS_points,A,B)

####Save environnment####

save.image()
